# Molecular Characteristics of Sweet Syndrome: A Systematic Review

**DOI:** 10.1111/exd.70022

**Published:** 2024-12-20

**Authors:** Laura Calabrese, Maurizio Romagnuolo, Martina D'Onghia, Pietro Rubegni, Angelo V. Marzano, Chiara Moltrasio

**Affiliations:** ^1^ Department of Medical, Surgical and Neurological Sciences, Dermatology Section University of Siena Siena Italy; ^2^ Institute of Dermatology Catholic University of the Sacred Heart Rome Italy; ^3^ Department of Medical Biotechnologies University of Siena Siena Italy; ^4^ Dermatology Unit Fondazione IRCCS Ca' Granda Ospedale Maggiore Policlinico Milan Italy; ^5^ Department of Pathophysiology and Transplantation Università degli Studi di Milano Milan Italy

## Abstract

Sweet syndrome (SS), originally described as acute febrile neutrophilic dermatosis, is a rare inflammatory skin condition, considered the prototype of neutrophilic dermatoses. It is characterised by the sudden onset of well‐defined tender papules, plaques and nodules often accompanied by fever, neutrophilia and elevated markers of inflammation. Several variants have been described both clinically and histopathologically. Classifications include idiopathic, malignancy‐associated, and drug‐induced SS. The exact pathogenesis of SS is still unclear; however, recent findings have shed light on the role of dermal infiltrating neutrophils—in the context of innate immunity, and signalling pathways related to adaptive immunity. To critically analyse the current molecular landscape of SS and discuss the recent evidence supporting novel potential immune mediators and biological signalling pathways involved in SS pathogenesis. The methodology followed PRISMA guidelines and included two bibliographical databases, searching articles published until 17 December 2023. Titles, abstracts and full text were reviewed independently by two assessors, while other two investigators resolved any opinion differences. Of 3303 records identified through database search, 22 articles met the eligibility criteria for inclusion. We considered experimental studies that performed molecular analysis, in terms of cytokines quantification, gene expression and/or immunofluorescence/immunohistochemistry. As for the latter, only studies aimed at characterising the nature of the inflammatory infiltrate and potential mechanisms leading to distinct forms of cutaneous inflammatory cell influx were included. Overall, we described research on 202 SS patients (177 skin biopsies and 25 blood specimens) revealing the predominant role of neutrophil activation and abnormal proliferation as unifying mechanisms in different SS subtypes. Interestingly, we found that hyperactivation of the IL‐1 pathway might occur only in a subset of SS patients and adaptive immunity could also play a role in the pathogenic scenario of SS, with a potential significant role of IL‐17 axis. This systematic review provides a wealth of evidence on the molecular landscape of SS, although further research is needed to a deeper understanding of the patho‐mechanisms of this rare disease and hopefully lead to targeted therapeutic approaches.

## 1 Introduction

Sweet syndrome (SS) is a rare inflammatory skin disease, originally described as ‘acute febrile neutrophilic dermatosis’ [1], characterised by the abrupt onset of well‐defined, painful, tender, erythematous papules, plaques, and nodules with a dense dermal neutrophilic infiltrate on histology, often accompanied by fever, leukocytosis with neutrophilia and elevation of serum inflammatory markers [2, 3].

SS is often reported in association with a broad range of comorbidities, leading to its classification into three main subtypes: (i) classical (idiopathic), often associated with infections, inflammatory bowel diseases (IBDs), pregnancy and autoimmune disorders; (ii) malignancy‐associated, correlated with both solid‐organ and hematologic malignancies (in particular acute myeloid leukaemia and myelodysplasias) and (iii) drug‐induced [4, 5]. Regarding the latter setting, SS has also been reported to follow COVID‐19 vaccines [6, 7].

In the absence of relevant comorbidities, SS is usually responsive to systemic corticosteroids; topical or intralesional corticosteroid injections may also be used, especially in localised forms [8]. For severe or refractory disease, several steroid‐sparing agents as well as biologic therapies such as interleukin (IL)‐1 blockers or tumour necrosis factor‐α (TNF‐α antagonist) have proven effective [9, 10]; however, literature evidence is still scarce and further research is needed to provide valuable insights into the complex pathogenesis of SS, paving the way for promising novel therapeutic strategies.

In actual fact, the exact cellular and molecular mechanisms involved in SS remain to be elucidated, although a unifying mechanism seems to be represented by an abnormal activation, proliferation, maturation and skin homing of neutrophils [11]. Cytokine dysregulation has also been proposed as a key factor that contributes to disease onset and progression. In classic SS, a serum and/or dermal overexpression of T helper type 1 (Th1)‐related cytokines seem to be responsible for neutrophil activation and accumulation, along with a suppression of Th2 cells. A role for granulocyte colony‐stimulating factor (G‐CSF) is enthralling since increased levels of the latter have been proposed as a potential factor in all three main SS subtype [11].

This systematic review aims to critically analyse the current molecular landscape of SS and discuss the recent evidence supporting novel immune mediators and biological signalling pathways involved in SS pathogenesis.

## 2 Methods

### 2.1 Search Strategy

MedLine (via PubMed) and Web of Science (WOS) databases were searched up to December 17, 2023. The main search in MedLine was performed using the string “(“Sweet syndrome” OR “Acute febrile neutrophilic dermatosis”)”. The main search in WOS was performed using the string “[TS=(“Sweet syndrome” OR “Acute febrile neutrophilic dermatosis”]”. Additionally, relevant keywords were used in different combinations for free‐hand search and bibliography of selected articles was reviewed. The search was designed and performed by two authors independently (MD and MR), under supervision of senior investigators (LC, CM). No date restriction was applied. We followed the Preferred Reporting Items for Systematic Reviews and Meta‐Analyses (PRISMA) guidelines for preparing our manuscript [12]. Besides, the present systematic review has been registered in the Prospective Register of Systematic Reviews (PROSPERO) database with the registration number CRD42024497714.

### 2.2 Study Eligibility Criteria

Only articles written in the English language were considered eligible. To be included in the final review, studies had to be published as full‐text original articles in international, peer‐reviewed journals. Prospective or retrospective cohort studies, cross‐sectional studies, case series, case report and letters were deemed eligible. The population, intervention, comparator, outcome (PICO) framework was used to build the search question. All studies meeting the following criteria were included in the final review:

- Population: patients with SS;
- Intervention: no intervention;
- Comparison: healthy controls or no control group depending on study design
- Outcome: experimental studies that performed molecular analysis on SS specimens, such as serum cytokines quantification, gene expression and/or immunofluorescence/immunohistochemistry studies. As for the latter, only studies aimed at characterising the nature of the inflammatory infiltrate with a direct implication on the in‐depth knowledge of SS pathophysiology were included. Ineligible studies included articles on conventional histology, genetic analysis, animals only or those that were a review of previously published data with no new Supporting Information.

### 2.3 Study Selection Process and Quality Assessment

**FIGURE 1:**
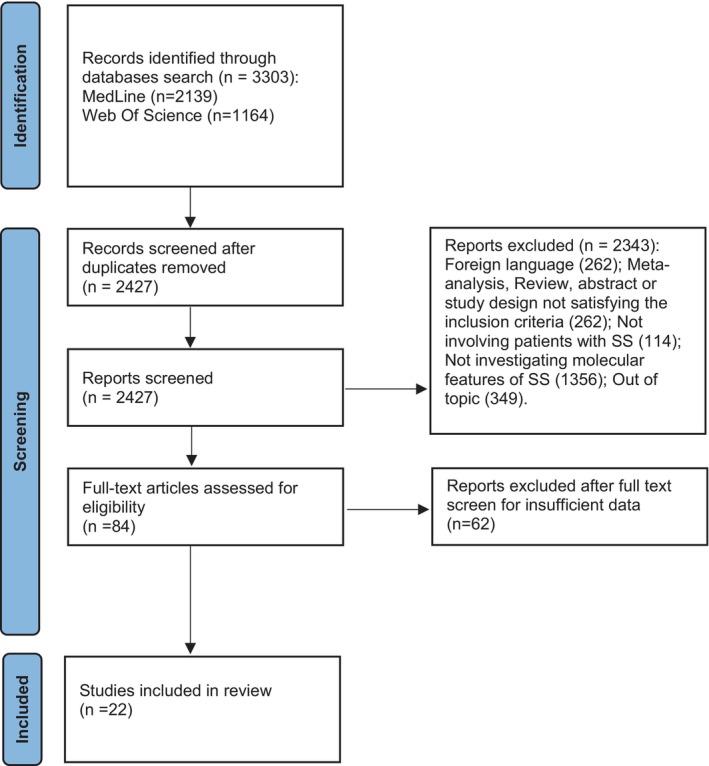
PRISMA_2020_flow_diagram. SS, Sweet syndrome [12].

After removal of duplicate records, two reviewers (MD and MR) independently screened titles and abstracts for the first‐step evaluation. Following the screening phase, the same two reviewers independently evaluated the full text of the remaining articles to determine eligibility for inclusion in the final review. Disagreements among the reviewers were resolved by discussion with the other senior investigators (LC and CM) until reaching a final consensus. Detailed flowchart of the study selection process is reported in Figure 1. The quality of studies was evaluated using the NIH quality assessment tool for Observational Cohort and Cross‐Sectional Studies and NIH Quality Assessment Tool for Case Series Studies [13] (Tables S1 and S2).

## 3 Results

### 3.1 Results of the Systematic Search

The search strategy identified 2139 records in PubMed and 1164 in WOS. After removal of duplicates, a total of 2427 studies proceeded to review. Of these, 2343 were excluded following screening titles and abstracts, and full text evaluation was performed on 84 articles. A total of 22 articles were deemed eligible for inclusion in qualitative synthesis (Figure 1).

### 3.2 Demographic and Clinical Features

Details of demographic and clinical characteristics of the patients included in the study are summarised in Tables 1 and 2. 202 SS patients, with a mean age of 50, 72 years (standard deviation +/−15,75, age ranging from 34 months to 93 years) were identified, with 104 (52%) of them being females, 79 (39%) males and 19 (9%) with no gender specified. Overall, 177 skin biopsies and 25 blood specimens were analysed in the studies. The clinical subtype of classical SS was specified in 72 patients, with 60 (84%) being idiopathic SS, 11 (15%) malignancy‐associated SS, and 1 drug‐induced SS (1%). Histiocytoid variant of SS (H‐SS) was reported in 99 patients belonging to four studies; in this group, 70 (71%) patients had an idiopathic H‐SS while 29 (29%) were malignancy‐associated. Overall, in nearly half of the patients (87 patients, 43%) one or more comorbidity was reported. Regarding treatment, systemic steroids and other immunosuppressive agents have been administered in most cases, with good clinical outcome (42% complete remission rate).

**TABLE 1:** Demographic characteristics of patients with Sweet syndrome included in the studies.

Author (year)	Country	N° patients	Sex	Mean Age (years)	N° controls
Reuss‐Borst et al. (1993)	Germany	1	F	Na	Na
Bourke et al. (1996)	England	12	Na	Na	12
Giasuddin et al. (1998)	Lybia	8	F	Na	11
Hattori et al. (2003)	Japan	1	M	Na	Na
Kawakami et al. (2004)	Japan	12	5 M; 7 F	43	10
Requena et al. (2005)	Spain, Germany	41	15 M; 26 F	56	Na
Corazza et al. (2008)	Italy	6	4 M; 2 F	52	Na
Uhara et al. (2008)	Japan	1	M	Na	Na
Marzano et al. (2010)	Italy	8	3 M; 5 F	Na	20
Marzano et al. (2011)	Italy	7	3 M; 4 F	Na	20
Marzano et al. (2014)	Italy	6	3 M; 3 F	44	6
Antiga et al. (2015)	Italy	5	3 M; 2 F	Na	9
Imhof et al. (2015)	Switzerland	1	M	Na	Na
Magro et al. (2015)	USA	13	8 M; 5 F	60	Na
Peroni et al. (2015)	Italy	12	7 M; 5 F	48	Na
Alegrìa‐Landa et al. (2017)	Spain, Austria, Germany	33	13 M; 20 F	46	Na
Laurisden et al. (2017)	USA	7	3 M; 4 F	59	7
Takano et al. (2017)	Japan	1	F	Na	Na
Matsuzawa et al. (2019)	Japan	1	M	Na	Na
Stalder et al. (2022)	Switzerland	8	4 M; 4 F	57	11
Bhattacharya et al. (2023)	USA	1 7	F Na	51 Na	13 13
Calabrese et al. (2023)	Italy	10	4 M; 6 F	66	12

Abbreviations: F, female; M, male; Na, not available.

**TABLE 2:** Clinical characteristics of patients with Sweet syndrome included in the studies.

Author, (year)	SS variant	Clinical subtype	Lesional site	Comorbidities	SS current treatment	Follow‐up
Reuss‐Borst et al. (1993)	Classical	Malignancy‐associated	Trunk, extremities	Myelodysplastic syndrome	CS; azathioprine; cyclophosphamide	CR
Bourke et al. (1996)	Classical	Na	Na	None	Na	Na
Giasuddin et al. (1998)	Classical	Idiopathic	Na	None	None	Na
Hattori et al. (2003)	Classical	Malignancy‐associated	Na	Myelodysplastic syndrome of the refractory anaemia type with trisomy 8	CS; potassium iodide	CR
Kawakami et al. (2004)	Classical Classical	Idiopathic Idiopathic	Na	None	Colchicine; CS; potassium iodide; dapsone; immunosuppressive agents	CR
Requena et al. (2005)	Histiocytoid	Idiopathic; malignancy associated	Neck, trunk, extremities	Chronic monocytic leukaemia; conjunctivitis; eyelid edema; erythema nodosum; monoclonal gammopathy of undetermined significance; diabetes mellitus; renal carcinoma; breast carcinoma; colitis ulcerosa; B cell chronic lymphocytic leukaemia; multiple myeloma	CS; NSAIDs	CR
Corazza et al. (2008)	Classical	Idiopathic; malignancy‐associated	Head, neck, extremities	Pharyingitis, parodontal disease, myelodysplasia; chronic myelo‐monocytic leukaemia; diffuse large B cell lymphoma; Hodkgin's lymphoma	CS	CR
Uhara et al. (2008)	Classical	Malignancy‐associated	Head, trunk, buttock	Behçet's disease; neutrophilic panniculitis; acute myeloid leukaemia	CS	CR
Marzano et al., 2010)	Classical	Idiopathic; malignancy‐associated	Na	B‐cell lymphocytic leukaemia	Na	Na
Marzano et al. (2011)	Classical	Idiopathic; malignancy‐associated	Na	B‐cell lymphocytic leukaemia	None	Na
Marzano et al. (2014)	Classical	Idiopathic; malignancy‐associated	Head, neck, trunk, extremities	B‐cell lymphocytic leukaemia	None	Na
Antiga et al. (2015)	Classical	Na	Na	None	None	Na
Imhof et al. (2015)	Classical	Drug‐induced (Azathioprine)	Trunk	Indeterminate colitis	Sulfasalazine; CS; Azathioprine	CR
Magro et al. (2015)	Histiocytoid	Idiopathic; malignancy associated	Head, neck, trunk, back, extremities	Acute myeloid leukaemia; myelodysplastic syndrome; myeloproliferative neoplasms; endometrioid adenocarcinoma; chronic joint pain; multiple myeloma; familial mediterranean fever	Prednisone; thalidomide; NSAIDs	CR
Peroni et al. (2015)	Histiocytoid	Idiopathic; malignancy associated	Head, trunk, extremities	Upper respiratory tract infection; Suspect Kawasaki disease; mielodysplasia; monoclonal gammopathy	CS; colchicine; methotrexate; acetlysalicylic acid; intravenous immunoglobulins	CR
Alegrìa‐Landa et al. (2017)	Histiocytoid	Na	Head; trunk; extremities	Myelodysplasia; chronic myelogenous leukaemia; acute myelogenous leukaemia, lung cancer	Na	Na
Laurisden et al. (2017)	Classical	Na	Na	None	Na	Na
Takano et al. (2017)	Classical	Idiopathic	Extremities	None	CS, colchicine	CR
Matsuzawa et al. (2019)	Classical	Malignancy‐associated	Extremities	Mielodysplasia; pulmonary toxoplasmosis	CS	Na
Stalder et al. (2022)	Classical	Idiopathic	Na	None	Na	Na
Bhattacharya et al. (2023)	Classical	Idiopathic	Head, neck, trunk, extremities	Primary sclerosing cholangitis	Anakinra	CR
Calabrese et al. (2023)	Classical	Idiopathic	Extremities	Behçet's disease; fibromyalgia; erythema nodosum; mixed connective tissue disease	CS	CR

Abbreviations: CS, corticosteroids; CR, complete remission; NSAIDs, nonsteroidal anti‐inflammatory drugs; Na, not available.

### 3.3 Inflammatory Infiltrate in SS: Classic vs. Histiocytoid SS

Immunohistochemical studies confirmed that dermal neutrophils represented the predominant infiltrating cells in lesional tissue (Table 3); occasionally, an admixed scattered infiltrate of macrophages and histiocytes expressing CD68/PMG1 [14, 15], T lymphocytes with significant levels of CD40/CD40 ligand [16, 17] and CD56^+^ natural killer (NK) cell [16] were also observed. By contrast, histiocytoid SS was characterised by myeloperoxidase (MPO)‐positive immature mononuclear cells [18], an abundance of CD68^+^ (PMG1) and M2‐like CD163^+^ macrophages [19, 20]. Subsequently, Alegrìa‐Landa et al. [21] pointed out that the majority of infiltrating cells belonged to the myeloid lineage, whereas scattered CD163^+^ cells should only be considered accompanying inflammatory cells. By contrast, Stadler and colleagues [22] reported abundant M2 macrophages in 4 SS lesional tissues (1 H‐SS and 3 classical SS).

**TABLE 3:** Molecular characterisation of patients with Sweet syndrome included in the studies.

Author (year)	Method of assessment	Tissue analysed	Stage of disease	Measurement method	Molecules analysed	Results
Reuss‐Borst et al. (1993)	Enzyme‐linked immunosorbent assay	Serum	Active	Serum concentration	IL‐1β; IL‐6; G‐CSF; GM‐CSF; IFN‐γ; TNF‐α	The levels of IL‐6 (4988 pg/mL) and G‐CSF (> 10 000 pg/mL) were found to be markedly increased while those of GM‐CSF (8 pg/mL) and TNF‐α (58 pg/mL) only slightly
Bourke et al. (1996)	Immunohistochemistry	Lesional	Na	Semi‐quantitative–intensity of staining	IL‐1α, IL‐1β, CD20, CD3, HLA‐DR, CD68, CD68/PGM1, CD11b, Neutrophil elastase	Neutrophils predominated while macrophages scattered throughout the dermis. Histiocytes were also present, although not predominantly A decreased staining for IL‐1α and IL‐1β was observed
Giasuddin et al. (1998)	Enzyme immunoassay	Serum	Active	Serum concentration	IL‐1α; IL‐1β; IL‐2; IFN‐γ; IL‐4	Significantly higher serum levels of IL‐1 (α and β), IL‐2, and IFN‐γ (*p* < 0.05) were found in SS patients versus healthy controls. IL‐4 levels were normal
Hattori et al. (2003)	Na	Serum	Active, inactive stable	Serum concentration	IL‐1β; IL‐6; G‐CSF	Serum levels of IL‐6 and G‐CSF were elevated in the active phase of disease (IL‐6 = 198 pg/mL; G‐CSF = 390 pg/mL) vs. the inactive phase (IL‐6 = 3 pg/mL; G‐CSF = 14 pg/mL). IL‐1β levels were not elevated throughout the disease course (< 10 pg/mL)
Kawakami et al. (2004)	Enzyme immunoassay	Serum	Active, inactive	Serum concentration	G‐CSF	G‐CSF levels (*p* < 0.05) were significantly higher in active SS when compared with the inactive stage of the disease
	Propidium iodide	Neutrophil fraction of peripheral blood		Flow cytometry	Rate of neutrophils apoptosis	The percentage of apoptotic nuclei (*p* < 0.05) in the neutrophils of patients with active SS was significantly higher at 18 h of incubation when compared with those of healthy controls
Requena et al. (2005)	Immunohistochemistry	Lesional	Active	Semi‐quantitative‐cell count (×600)	CD15; CD34; CD43, CD45 (LCA); CD45RO; CD66; CD68; HAM56; Lysozyme; MAC387; MPO; NE; TIA‐1	Inflammatory infiltrate showed the pan‐histiocytic markers CD68, HAM‐56, MAC‐386, and lysozyme. A strong immunoreactivity for MPO in most of the mononuclear cells of the dermal infiltrate was found
Corazza et al. (2008)	Immunohistochemistry	Lesional	Active	Qualitative‐intensity of staining	MPO; CD68/PGM1	The inflammatory infiltrate was mainly composed of neutrophils in 3 cases, histiocytes in 1 case and mixed neutrophils and histiocytes in two cases. In all the cases the inflammatory infiltrate was composed of MPO^+^ and CD68/PGM1^+^ cells
Uhara et al. (2008)	Radioimmunoassay	Serum	Active, inactive	Serum concentration	G‐CSF	G‐CSF serum levels were elevated during active disease (294 pg/mL) compared to inactive disease (32 pg/mL), although statistical significance was not reported
Marzano et al. (2011)	Immunoistochemistry	Lesional	Active	Semi‐quantitative–cell‐count (×200)	CD3; CD163; MPO; TNFα; IL‐17; IL‐8; MMP2; MMP9; VEGF	Protein levels of MPO (*p* = 0.014), IL‐8 (*p* = 0.001), MMP9 (*p* = 0.035) were significantly higher in SS lesional skin vs. HCs. CD3, CD163, TNF‐α, IL‐17, MMP2, VEGF showed a trend towards upregulation, although these differences were not statistically significant
Marzano et al. (2010)	Immunoistochemistry	Lesional	Active	Semi‐quantitative—cell‐count (×200)	CD3; CD163; MPO; TNFα; IL‐17; IL‐8; MMP2; MMP9; VEGF	Protein levels of CD3, CD163, MPO, TNF‐α, IL‐17, IL‐8, MMP2, MMP9, VEGF were all significantly higher in lesional SS skin samples, vs. HCs (*p* = 0.0001)
Marzano et al. (2014)	Homogenisation of lesional skin with RIPA buffer containg protease‐ and phosphatase inhibitors	Lesional	Active	Cytokine antibody array	IL‐1β, IL‐1RI, IL‐1RII, TNF‐α, TNF‐RI, TNF‐RII, IL‐17, IL‐17R, L‐Selectin, IL‐8, CXCL1,2,3, CXCL16, RANTES, MMP2, MMP9, TIMP‐1, TIMP‐2, Siglec 5, Siglec 9, Fas, FasL, CD40, CD40L	Protein levels of IL‐1β (*p* = 0.004), IL‐1R1 (*p* = 0.04), TNF‐α (*p* = 0.02), IL‐17 (*p* = 0.011), IL‐17R (*p* = 0.025), L‐selectin (*p* = 0.029), IL‐8 (*p* = 0.018), CXCL1,2,3 (*p* = 0.006), CXCL16 (*p* = 0.036), MMP2 (*p* = 0.02), TIMP‐1 (*p* = 0.007), Siglec 5 (*p* = 0.001), Siglec 9 (*p* = 0.001), Fas (*p* = 0.047), FasL (*p* = 0.008), and CD40L (*p* = 0.021) were significantly higher in lesional SS skin samples compared to HCs. A Additionally, protein levels of IL‐1RII, TNF‐RI, TNF‐RII, RANTES, MMP9, and TIMP‐2 showed a trend towards upregulation, although these differences were not statistically significant
Antiga et al. (2015)	Immunoistochemistry	Lesional	Active	Semi‐quantitative‐cell‐count (×400)	IFN‐γ, IL‐12, CXCR3, CCR5, IL‐4, IL‐5, IL‐13, CCR3, CD40, CD40L, IL‐15, CD56	Protein levels of IFN‐γ, IL‐12, CXCR3, CCR5, IL‐4, IL‐5, IL‐13, CCR3, CD40, CD40L, IL‐56 and CD56 were all significantly higher in lesional SS skin samples, vs. HCs (*p* = < 0.05)
Imhof et al. (2015)	mRNA expression	Lesional	Active	Real‐time PCR	IL‐1β	IL‐1β expression levels, in lesional skin, were over 250‐fold higher than those observed in unaffected skin
	Immunoistochemistry	Lesional	Active	Qualitative‐intensity of staining	IL‐1β	Expression of the mature active form of IL‐1β was enhanced in inflammatory cells of the dermal infiltrate, mainly in neutrophils
Magro et al. (2015)	Immunistochemistry	Lesional	Active	Semi‐quantitative—cell‐count (×100)	CD4; CD163; CD68; CD16; CD14; HLA‐DR; chloroacetate esterase; MPO, CD123; TCL1‐oncogene; MXA; BDCA‐3	Neutrophils were sparce or absent. Dermal infiltrating histiocytoid cells strongly expressed CD68 (PG‐M1) (80/90%), CD163 (80/90%), MPO (from 30% to 90%) Variable positivity for myeloid dendritic cell markers such as CD11c, BDCA‐3, TCL1 oncogene, MXA and CD123 was observed
Peroni et al. (2015)	Immunohistochemistry	Lesional	Na	Semi‐quantitative—cell‐count (×100)	CD1a; CD3; CD15; CD20; CD34; CD68/KP1; CD68/PGM1; CD117; CD163; factor XIIIa; MPO	Most infiltrating cells expressed macrophage markers [CD68 (40%–50%), CD163 (~40%)] with a minority of lymphocytes [mostly T cells (~30%)] and sparse mature neutrophils [CD15 (~10%)]. Histiocytoid cells showed immunoreactivity for CD68 [with similar intensity for KP1 (~40%) and PG‐M1 (~45%) in all but 5 cases, which showed stronger reactivity for PG‐M1], MPO, CD163, and myeloid cell nuclear differentiation antigen, and were CD15‐, CD34‐, and CD117
Alegrìa‐Landa et al. (2017)	Immunohistochemistry	Lesional	Active	Semi‐quantitative—Cell‐count (×20–×400)	CD34; c‐kit/Cd117; MPO; CD163; CD14; MNDA	Most infiltrating mononuclear cells were MPO positive supporting the immature myeloid nature. 10%–25% of CD163 cells were peripherally distributed and should be interpreted as accompanying inflammatory cells
Laurisden et al. (2017)	Immunoistochemistry	Lesional	Active	Semi‐quantitative‐intensity of staining	Collagen I, Collagen IV, Laminin, Fibronectin, MPO	The protein levels of collagen I (*p* < 0.05) and collagen IV (*p* < 0.00001) were significantly elevated in SS skin samples compared to HCs MPO staining was also increased in SS samples relative to healthy controls, although statistical significance was not reported The expression of laminin and fibronectin was correlated with MPO intensity, showing a strong correlation for laminin but not for fibronectin. However, comparisons with HCs for laminin and fibronectin expression were not conducted
Takano et al. (2017)	Cytokine array	Serum	Active	Na	IL‐6, IL‐1β, neopterin, Il‐18, sTNF‐RI, sTNFr‐II	Elevated levels of IL‐6 (48 pg/mL) before therapy with systemic steroids, decreased to normal range (< 5 pg/mL) 5 weeks after therapy. Levels of IL‐1β, IL‐18, neopterin, and sTNF‐R I and II were not elevated before treatment
Matsuzawa et al. (2019)	Cytokine array	Serum	Active	Bead‐based immunoassay	IL‐6, IL‐10, IL‐2, IL‐4, IL‐17A, TNF, IFN‐γ	Elevation of IL‐6 (448 pg/mL) and IL‐10 (10,11 pg/mL) during the active phase of disease in comparison to inactive phases [IL6 < 2 pg/mL; IL‐10 < 7 pg/mL]
Stalder et al. (2022)	Immunofluorescence	Lesional	Active	Semiquantitative‐cell count (×40)	IL‐17E; iNOS; arginase 1 in combination with MPO and CD68	An increased expression of IL‐17E and iNOS was observed on epidermal keratinocytes. Epidermal neutrophils exhibited an MPO^+^CD68^+^IL‐17E low signature. Immature neutrophils and antiinflammatory macrophages were very common
Bhattacharya et al. (2023)	mRNA expression	Lesional	Active	Microarray profiling	Total RNA, expression quantified for targeted genes	Microarray profiling of refractory SS vs. HCs showed a significant upregulation of IL‐1β gene (FC: 2.09; *p* < 0.001) and downregulation of PIK3R1 (FC: −4; *p* < 0.001). The top 10 DEG were: SRGN (FC: 352.82; *p* < 0.001), CXCL9 (FC: 246.4; *p* < 0.001), TIMP1 (FC:123.8; *p* < 0.001), AC245036.6 (FC: 82.81; *p* < 0.001), CHI3L2 (FC: 51.39; *p* < 0.001), CD163 (FC: 47.43; *p* < 0.001), S100A9 (FC:46.63; *p* < 0.001), LYZ (FC: 45.77; *p* < 0.001), AC245036.5 (FC: 45.53; *p* < 0.001), AC245036.4 (FC: 45.53; *p* < 0.001)
	mRNA expression	Lesional	Active	Real‐time PCR	IL‐1β	IL‐1β transcript was significantly increased in SS refractory patient vs. HCs (*p* < 0.01)
	Protein levels	Lesional	Active	Western blot	p‐AKT, IL‐1R1	Neutrophils from the patient with refractory SS exhibited increased AKT activation and IL‐1R1 expression
	Gene expression	Lesional	Active	Microarray profiling	IL‐1β	Increased IL‐1β transcript levels were detected in the SS refractory patient's compared with levels in HCs (*p* < 0.001) Individual samples from patients with SS exhibited varied levels of IL‐1β transcripts. Three patients had transcript levels greater than 2.5 standard deviations above the average for the HCs
Calabrese et al. (2023) 37 724 787	Immunoistochemistry	Lesional	Active	Semi‐quantitative—Cell count (×200)	IL‐1β; IL‐36γ; IL‐33; IL‐1R3	Marked overexpression of dermal IL‐1β (*p* < 0.001) and IL‐1R3 (*p* = 0.02) was found
	Immunofluorescence	Lesional	Active	Semi‐quantitative—Cell count (×400)	IL‐1β/MPO; IL‐1β/CD68	Co‐expression patterns of IL‐1β showed that it was secreted mostly by neutrophils, but also to a lesser extent by macrophages
	In situ hybridisation	Lesional	Active	Semi‐quantitative‐cell count (×100; ×400)	IL‐1β; IL‐36γ; IL‐33; IL‐1R3	Marked overexpression of dermal IL‐1β (*p* < 0.001) and IL‐1R3 (*p* = 0.02) was reported

Abbreviations: BDCA‐3, blood dendritic cell antigen 3; CCR, chemokine receptors; CD, cluster of differentiation; CXCL, chemokine C‐X‐C motif ligand; CXCR, C‐X‐C chemokine receptor; FASL, fas ligand; G‐CSF, granulocyte‐colony stimulating factor; GM‐CSF, granulocyte‐macrophage colony‐stimulating factor; HAM56, human alveolar macrophage; HLA‐DR, human leukocyte antigen–DR isotype; IFN, interferon; IL, interleukin; IL‐xR, interleukin receptor; iNOS, inducible‐NO synthase; MAC387, clone of macrophages/monocytes/granulocytes antibody; MMP, matrix metalloproteinases; MNDA, myeloid cell nuclear differentiation antigen; MPO, myeloperoxidase; MXA, myxovirus protein; Na, not available; NE, neutrophil elastase; p‐AKT, phosphorylated Akt; PGM1, clone anti‐CD68; RANTES, regulated on activation, normal T‐cell expressed and secreted; Siglec, sialic‐acid‐binding immunoglobulin‐like lectins; sTNF‐R, soluble tumour necrosis factor receptor; TCL1, T‐cell leukaemia/lymphoma 1; TIA1, T‐cell intracytoplasmic antigen; TIMP, tissue inhibitors of matrix metalloproteinases; TNF, tumour necrosis factor; TNF‐R, tumour necrosis factor receptor; VEGF, vascular endothelial growth factor.

### 3.4 Autoinflammation and Th1/Th2/Th17 Polarisation

The majority of the included studies pointed out the pivotal role of autoinflammatory pathways in the pathophysiology of SS (Table 3). IL‐1β was found in elevated concentrations both in serum and lesional tissue of most SS patients [15, 17, 23, 24, 25], as well as the co‐receptor IL‐1R3, although the latter only in SS lesional skin [25]. Noteworthy, although an increased concentration of IL‐1β and its family members was not constantly found in SS serum and skin specimens [26, 27, 28], their downstream effectors and induced cytokines, including granulocyte‐colony stimulating factor (G‐CSF) and IL‐6 were found in elevated concentrations in peripheral blood of SS patients, especially during the active stages of disease [26, 27, 28, 29, 30, 31]. TNF‐α and its receptor were also found over‐expressed in SS lesional tissue [17, 32, 33] and only in one case in serum [27], whereas normal levels of this cytokine were reported in two patients [28, 31].

Several other chemokines such as IL‐8 and chemokine (C‐X‐C motif) ligand (CXCL) 1/2/3 and 16, were found to be increased at lesional skin level, promoting an inflammatory process, which in turn was favoured by the up‐regulation of L‐selectin, FAS and Siglec (sialic‐acid‐binding immunoglobulin‐like lectins) 5/9 [17]. In addition, other inflammatory mediators, such as metalloproteinases (MMPs), mainly MMP‐9 and MMP‐2 [17, 32, 33] and VEGF (vascular endothelial growth factor) [32, 33], were found overexpressed in SS lesional tissue.

Finally, other Th1 inflammatory markers such as IL‐12 and IL‐15, interferon (IFN)‐γ, C‐X‐C chemokine receptor (CXCR) 3 and C‐C chemokine receptor type (CCR) 5 were found upregulated in SS lesional tissue [16], with the only exception of IFN‐γ, which was also upregulated in serum [24].

As regard to IL‐17 and its receptor (IL‐17R) were also reported upregulated in SS lesional tissue; interestingly, an overexpression of IL‐17E and inducible nitric oxide synthase (iNOS) was detected in the epidermis of SS skin samples [22]. Overall, the pathogenesis of SS appears to be predominantly characterised by the Th1 signature, although the presence of a Th2 signature with modestly increased expression of IL‐4, IL‐5 and IL‐13 has also been reported [16].

### 3.5 Gene Expression Studies

A recent study of Bhattacharya et al. [34] reported 1.622 differentially expressed genes (DEGs) in the patient's SS lesional skin compared with HCs, revealing an enrichment for genes involving neutrophil‐specific functions as well as neutrophil‐related immune pathways and regulation of IL‐1 production. As for the latter, IL‐1β gene expression was substantially increased at the lesional skin level of a subset of SS patients, differently from what was observed for neutrophil marker genes such as MPO and arginases.

## 4 Discussion

Since its first identification in 1964, our knowledge of SS has broadened, and novel clinical and histologic variants have emerged. It is now accepted that the remarkable clinical heterogeneity of the disease, the existence of different subtypes, as well as the wide variety of associated conditions mirror the pathogenetic complexity of SS that can in no way be encapsulated in a single paradigm.

The disease's heterogeneity is even reflected in the immune cell infiltration landscape. Immunohistochemical studies included in our review confirmed the predominance of neutrophils in most SS specimens, as well as the heterogeneity in other cell populations, consistent with the existence of different histotypes. Surely, one histologic variant that is still poorly characterised and stands out from the others is H‐SS, whose origin, myeloid [35, 36] or histiocytic [19, 20, 37] has been debated. The H‐SS cell infiltrate was originally described as mostly composed of histiocytoid appearing mononuclear cells, then interpreted as immature neutrophils based on the marked MPO expression [18]. Subsequently, Peroni et al. [19], and Magro et al. [20] reported infiltrating histiocytoid cells expressing CD68 (PG‐M1), MPO and CD163. The latter is considered a specific marker for M2‐like macrophages, mainly involved in non‐inflammatory responses, and acting as chemokine scavengers [38]. This finding led both groups of authors to suggest that H‐SS could be the expression of a cutaneous infiltration of M2 macrophages, with a representative concomitant MPO expression. Two following studies highlighted the immature myeloid nature of the infiltrate [21, 22]. In the former work, the authors interpreted the scattered and predominantly periphery macrophages CD163^+^ as accompanying inflammatory cells [21], whereas in the second one, abundant M2‐macrophages with regulatory functions were detected [22]. In light of the studies reviewed, whether the landscape of infiltrating immune cells represents mere bystanders or reflects a different stage of disease or a specific molecular signature for this histological entity, is yet to be clarified.

Interestingly, most of the included studies pointed out the pivotal role of autoinflammation in SS, as evidenced by previous studies on the pathogenic scenario of neutrophilic dermatoses, including SS [39, 40]. Autoinflammation primarily involves a dysregulation of innate immune response characterised by amplification loops of IL‐1 cytokine production and IL‐1 family pathways, including IL‐36 [41, 42]. IL‐1β, the active form of IL‐1, which is a marker of inflammasome activation with pleiotropic effects mainly on neutrophil‐mediated inflammation, was found to be overexpressed both at RNA and protein levels in most of the included studies conducted on skin samples of SS [15, 16, 17, 23, 25, 34]. Surprisingly, molecular studies focusing on the expression of the IL‐1 family on SS skin highlighted the often‐inhomogeneous expression of IL‐1β in different samples analysed, suggesting that hyperactivation of the IL‐1 pathway might actually occur only in a subgroup of SS patients. This concept becomes evident and even finds a possible explanation in the study conducted by Bhattacharya et al. [34], in which a gain‐of‐function *PIK3R1* pathogenic variant in neutrophils derived from a SS patient was found. This somatic variant led to an increased expression of IL‐1R1, cell migration towards IL‐1β, and neutrophil respiratory burst [34], suggesting that SS might be caused by a cell‐intrinsic and not cell‐extrinsic process and results from acquired mutations affecting signalling pathways that modulate neutrophil function. However, further experimental studies are needed to confirm this hypothesis. The authors also analysed IL‐1β transcript levels on other SS skin specimens and found increased expression only in 3 out of 7 samples, thus endorsing the hypothesis that increased IL‐1β signalling may exist in a subgroup and not in all SS patients. Similar results were shown in a study by our group, in which immunohistochemistry and in situ hybridization were performed on 10 SS samples, showing an increased expression of IL‐1β, compared to HCs‐, only in selected SS samples and even limited to some areas of intense dermal inflammation within one tissue section [25]. The heterogeneity observed in IL‐1β levels across SS samples may possibly reflect specific disease characteristics or patient‐related factors. For instance, IL‐1β expression could vary with disease severity and stage, as inflammatory intensity may correlate with symptom severity or disease burden. However, since SS is defined by the abrupt onset of cutaneous lesions, biopsies are typically taken at a similar stage, that is during the acute disease phase. Additionally, variability in the inflammatory response may be influenced by underlying conditions associated with different SS subtypes, though data on IL‐1β profiles specific to these subtypes remain limited. The same study further investigated other members of the IL‐1 family, such as IL‐36γ. It is well‐known that IL‐36 pathway is a driver of inflammation and when dysregulated can be associated with certain auto‐inflammatory dermatoses [43]. Our study did not report a significant overexpression of IL‐36γ in comparison to HCs [25] and this could be explained by the observation that IL‐36γ is an epidermal cytokine, predominantly produced by keratinocytes, and that SS is not usually featured by any significant epidermal changes. Therefore, in the specific case of SS, the role of IL‐36 in triggering the neutrophilic inflammation might be minor compared to other cytokines of the same family.

Although the expression of IL‐1 family members in SS sera was inconstant, a number of their downstream effectors and induced cytokines were instead found to be elevated in serum. Among these, IL‐6, whose role is supported by a recent report of refractory SS being managed with tocilizumab [44], has been shown to be overexpressed in SS serum, especially during the active stages of the disease [26, 28, 31]. G‐CSF, a haematopoietic growth factor able to increase the number of peripheral and active mature neutrophils [45], also acts as a major player on the SS pathophysiology and its role has been confirmed by our review. Indeed, most studies demonstrated increased G‐CSF serum levels, often related to active phase of the disease [26, 27, 30], suggesting that it may promote stimulatory effects on the neutrophils production and function. The role of G‐CSF in disease pathogenesis is further supported by the high frequency of drug‐induced SS resulting from its exogenous use, as well as by the possibility of both solid and haematological malignancies to produce G‐CSF, thus contributing to the onset and maintenance of malignancy‐associated SS [46]. Other mediators, such as TNF‐α, was found to be overexpressed in the SS lesional skin and, albeit inconstantly, in SS patients' serum [17, 27, 31, 32, 33]. Convincing evidence on the role of TNF‐α in SS stems from the successful use of TNF‐α blockers for the treatment of recalcitrant SS cases [10]. Furthermore, it can be hypothesised that TNF‐α and IL‐1β act synergistically in an inflammatory crosstalk by inducing adhesion molecules and chemoattractants such as IL‐8 (CXCL8), also upregulated in SS lesional skin [30, 32]. IL‐8 is probably one of the main responsible, alongside with G‐CSF, for the neutrophils recruitment in SS lesions. Several other chemokines, involved in both the recruitment of neutrophils (CXCL1, 2, 3) and NK and T cells (CXCL16, CXCL9, CXCR3, CCR5) were found to be overexpressed in SS lesional skin, either at protein or RNA level, suggesting that not only innate but also adaptive immunity plays a role in SS. Interestingly, several molecules involved in tissue remodelling were also found to be significantly overexpressed in SS skin in comparison to HCs. Those included MMP‐2, VEGF, collagen IV and TIMP‐1 [17, 32, 33, 47], the latter being counted among the top 10 DEGs in SS skin compared to HCs in one microarray profiling study [34]. These findings suggest that an active tissue remodelling is present in SS lesions and might be relevantly involved in inducing both tissue damage and repair in this disease. For example, VEGF, originally identified as an endothelial cell‐specific growth factor that stimulates angiogenesis, has recently been deemed responsible for increased vascular permeability, resulting in augmented leukocyte recruitment at the site of inflammation, which could be central for the development of SS lesions [48]. Another important insight from our review concerns the contribution of adaptive immunity in a disease classically considered purely autoinflammatory. An upregulation of inflammatory markers such as IL‐12, IL‐15 and IFN‐γ [16], corroborates a significant role of the Th1 response in the SS pathogenesis, which could even actively contribute to neutrophils recruitment in lesional tissue. In the context of adaptive immunity, a certain role of the IL‐17 pathway has emerged from our study as well. Indeed, both IL‐17 and its receptor as well as epidermal IL‐17E/iNOS were found to be overexpressed in SS lesional skin [16, 22, 32, 33]. IL‐17 cytokines, already associated with a plethora of immune‐mediated inflammatory skin diseases [49], exert a multitude of pro‐inflammatory effects, and in the pathogenic scenario of SS, could further increase IL‐17 production and immune cell infiltrations in a feed‐forward inflammatory loop. To date, only a single case report of refractory SS successfully treated with the IL‐17 inhibitor brodalumab has been described [50]. Finally, a Th2 signature with modestly increased expression of IL‐4, IL‐5 and IL‐13 has also been reported in SS skin in one study [16], while others reported no significantly increase of IL‐14 in SS serum [24, 31]. Therefore, the upregulation of Th2 mediators in SS is likely to be occasional and the role of this pathway rather marginal in the pathogenesis of this disease.

Our review has three main limitations; the first one is that the included studies did not analyse the clinical and histological SS variants separately; therefore, some clinical and molecular heterogeneity of samples in different studies or even in the same study is to be expected. The second one is that it was not always clear whether the patients were on a specific medication at the time the sample was taken, and this may have had a significant influence on the molecular profile. Finally, a wide range of technologies was adopted potentially making the results comparison less accurate.

## 5 Major Open Questions

While most research focused upon the autoinflammatory nature of SS and the contribution of neutrophils to disease activity, ongoing questions regarding the interrelationships between innate and adaptive immunity activity as well as the contribution of other immune cells such as lymphocytes and macrophages require further investigations. Furthermore, it has been reported that neutrophil extracellular traps (NETs) play an important role in the pathogenesis of various NDs [51]. However, it remains to be clarified how neutrophils generate NETs and what is the regulatory mechanism underlying potential NETs generation in SS. Finally, as bioinformatic gene expression profiling methods and genetic variants identification tools are increasingly emerging in the era of personalised medicine, findings on the role of genetic susceptibility and onset as well as the identification of specific biomarkers related to clinical phenotype, disease severity and treatment response will shed more light on the etio‐pathogenesis of SS.

## 6 Conclusions and Perspectives

**FIGURE 2:**
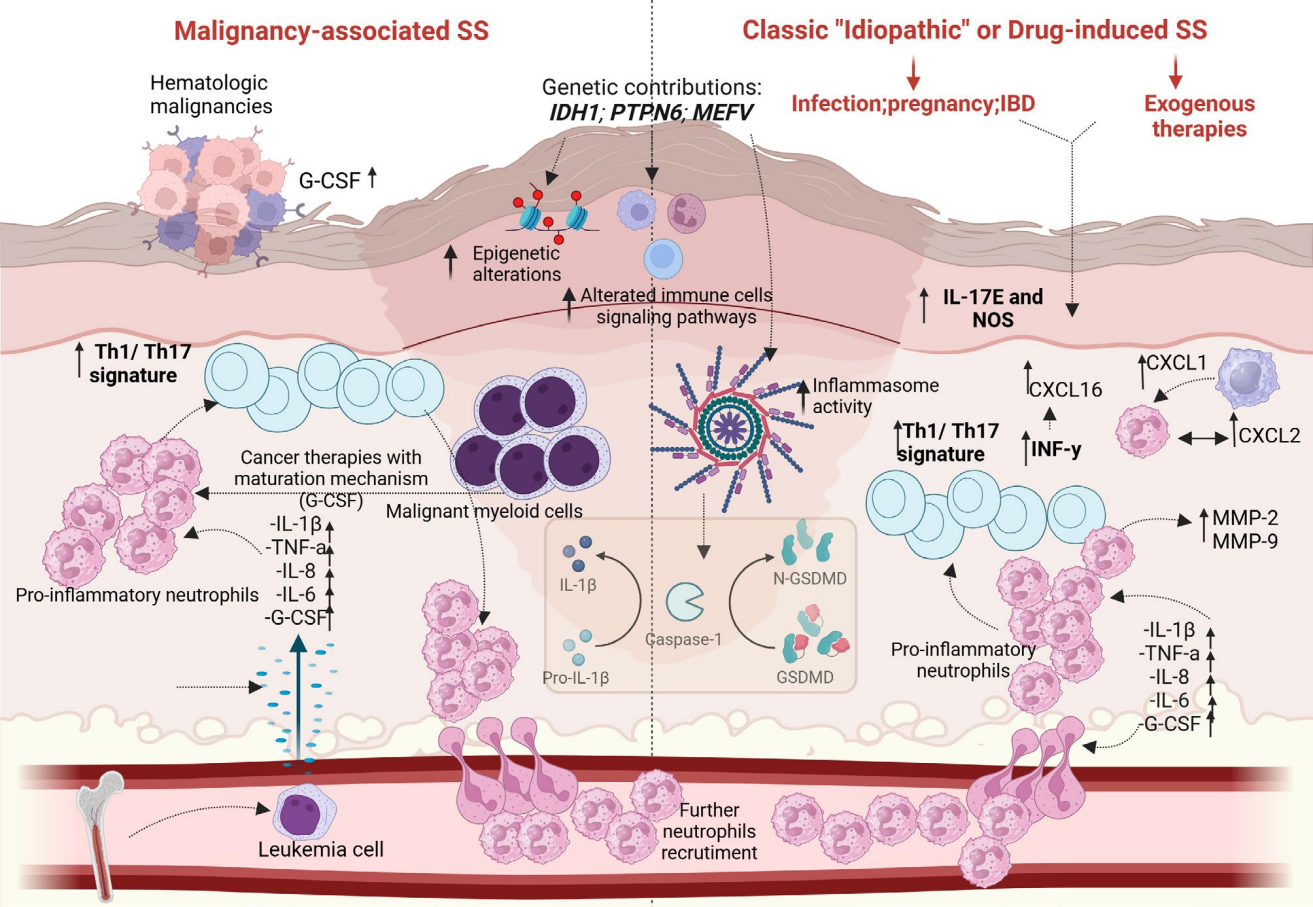
Main pathogenetic events in Sweet Syndrome based on the setting of occurrence.

In conclusion, despite the pathogenesis of SS is still incompletely elucidated, it seems clear that both neutrophil activation and abnormal proliferation are unifying mechanisms in different SS subtypes (Figure 2). Although it is now well established that SS is hallmarked by an autoinflammatory nature and is primarily driven by an innate immunity dysregulation, increasing evidence suggests that adaptive immunity could also play a role, although the exact axis, in particular between Th1 and Th17, as well as the extent of their involvement in the onset and maintenance of SS has not yet been precisely deciphered. Further molecular studies based on the application of new cutting‐edge technologies could allow a deeper understanding of the patho‐mechanisms of the disease and hopefully lead to targeted therapeutic approaches for SS in the future. Advances in research may allow for the identification of molecular signatures, potentially enabling targeted therapies that are tailored to the individual's molecular profile. Furthermore, combining therapies that target multiple inflammatory pathways (e.g., IL‐1 and TNF‐α inhibitors) may prove more effective than monotherapy, particularly in patients with refractory or recurrent disease. Personalised approaches that consider the specific inflammatory subtype of SS, as well as the presence of comorbidities, will be essential for optimising treatment outcomes and minimising side effects.

## Author Contributions

The topic of the manuscript was conceived by L.C. and M.R. The first draft of the manuscript was written by L.C., C.M., M.R., M.D. Schematics were prepared by M.D. and M.R. Finally, P.R. and A.V.M. revised and finalised the manuscript. All authors read and approved the final manuscript.

## Ethics Statement

The authors have nothing to report.

## Conflicts of Interest

The authors declare no conflicts of interest.

## Supporting information


Appendix S1.

## Data Availability

All data are incorporated into the article and the Supporting Information.
