# Supplementary material for: Molecular Characteristics of Sweet Syndrome: A Systematic Review
Source: Exp Dermatol. 2024 Dec 20;33(12):e70022. doi: 10.1111/exd.70022 (PMC11660222; doi:10.1111/exd.70022)
Supplement: Supplementary file 1 — Appendix S1. [file EXD-33-e70022-s001.docx]

**Table S1 Quality assessment of included studies according to NIH Quality Assessment Tool for Observational Cohort and Cross-Sectional Studies**

| **Author, year** | **1** | **2** | **3** | **4** | **5** | **6** | **7** | **8** | **9** | **10** | **11** | **12** | **13** | **14** |
| --- | --- | --- | --- | --- | --- | --- | --- | --- | --- | --- | --- | --- | --- | --- |
| Bourke et al., 1996 | Y | Y | Y | Y | N | N | Y | N/A | N/R | N/R | Y | N | N/A | N/A |
| Giasuddin et al., 1998 | Y | Y | Y | Y | N | N | Y | Y | Y | N | Y | N | N/A | N/A |
| Kawakami et al., 2004 | Y | Y | Y | Y | N | N | Y | Y | Y | N | Y | N | N | N/A |
| Requena et al., 2005 | Y | Y | Y | Y | N | N/A | N/A | N/A | Y | N/A | Y | N/A | N | N/A |
| Corazza et al., 2008 | Y | Y | Y | Y | N | N/A | Y | N/A | N/A | N/R | Y | N/R | N/A | N/A |
| Uhara et al., 2008 | Y | Y | N/A | N/A | N/A | Y | Y | N/A | N/A | Y | N/A | N | N/A | N/A |
| Marzano et al., 2010 | Y | Y | N/A | Y | N | N/A | N/A | N | Y | N | Y | Y | N/A | N/A |
| Marzano et al., 2011 | Y | Y | N/A | Y | N | N/A | N/A | N | Y | N | Y | Y | N/A | N/A |
| Marzano et al., 2014 | Y | Y | N/A | Y | N | N/A | N/A | N | Y | N | Y | Y | N/A | N/A |
| Antiga et al., 2015 | Y | Y | N/A | Y | N | N/A | N/A | N | Y | N | Y | Y | N/A | N/A |
| Peroni et al., 2015 | Y | Y | Y | Y | N | N | Y | N/A | Y | N | Y | N/R | N/A | N/A |
| Magro et al., 2015 | Y | Y | Y | Y | N | N/A | N/A | N/A | Y | N/A | Y | N/A | N | N/A |
| Alegrìa-Landa et al., 2017 | Y | Y | Y | Y | Y | N/A | Y | N/A | Y | Y | Y | N/A | N | N/A |
| Laurisden et al. 2017 | Y | Y | N/A | Y | N | N/A | N/A | N | N | N | Y | Y | N/A | N/A |
| Stalder et al., 2022 | Y | N | Y | Y | N | N | Y | Y | Y | N | Y | N | N | N/A |
| Bhattacharya et al. 2023 | Y | Y | N/A | Y | N | N/A | N/A | Y | Y | Y | Y | N | N/A | N/A |
| Calabrese et al., 2023 | Y | Y | Y | Y | N | N | Y | Y | Y | N | Y | N | N | N/A |

Legend: Y, Yes; N, no; N/A, not applicable; N/R, not reported

**Table S2 Quality assessment of included studies according to NIH Quality Assessment Tool for Case Series Studies**

| **Authors, year** | **1** | **2** | **3** | **4** | **5** | **6** | **7** | **8** | **9** |
| --- | --- | --- | --- | --- | --- | --- | --- | --- | --- |
| Reuss-Borst et al., 1993 | Y | Y | N/A | N/A | Y | N/A | N | N/A | Y |
| Hattori et al., 2003 | Y | Y | N/A | N/A | Y | N/A | Y | N/A | Y |
| Uhara et al., 2008 | Y | Y | N/A | N/A | Y | N/A | Y | Y | Y |
| Imhof et al., 2015 | Y | Y | N/A | N/A | Y | N/A | Y | N/A | Y |
| Takano et al., 2017 | Y | Y | N/A | N/A | Y | N/A | N | N/A | Y |
| Matsuzawa et al., 2019 | Y | Y | N/A | N/A | Y | N/A | N | N/A | Y |

Legend: Y, Yes; N, no; N/A, not applicable
